# Acute Disseminated Encephalomyelitis Following COVID-19 Infection

**DOI:** 10.7759/cureus.33365

**Published:** 2023-01-04

**Authors:** Farah Assi, Rim Abdallah, Ali Mecheik, Hassan H Rahhal, Jaafar Wazne

**Affiliations:** 1 Internal Medicine, Infectious Diseases, Lebanese University, Faculty of Medical Sciences, Beirut, LBN; 2 Allergy and Immunology, Internal Medicine, Lebanese University, Faculty of Medical Sciences, Beirut, LBN; 3 Intensive Care Unit, St. Georges Hospital - Hadath, Beirut, LBN; 4 Infectious Diseases, Bahman Hospital, Beirut, LBN; 5 Neurology, St. Georges Hospital - Hadath, Beirut, LBN

**Keywords:** pulsed-dose steroids, covid-19-induced ards, intravenous immunoglobulins, covid-19, sars-cov-2, acute disseminated encephalomyelitis

## Abstract

Acute disseminated encephalomyelitis (ADEM) is a relatively rare, post-inflammatory, immune-mediated demyelinating central nervous system disease that is predominantly reported in pediatric populations. Following the emergence of severe acute respiratory syndrome coronavirus 2, cases of ADEM are being reported following infection with this virus. Our case report describes a male patient in his early 40s who developed severe coronavirus disease 2019 (COVID-19) that rapidly progressed to a critical disease requiring invasive mechanical ventilation and high positive end-expiratory pressure, which was complicated by extensive neurological involvement and quadriplegia. MRI of the brain showed characteristic demyelinating lesions, suggestive of ADEM. As other entities were ruled out, our patient was treated using pulse steroids and intravenous immunoglobulins. The patient showed a good response to treatment and had an overall good prognosis, despite the severity of his condition. ADEM following COVID-19 is a rare entity worldwide.

## Introduction

Severe acute respiratory syndrome coronavirus 2 (SARS-CoV-2) has been reported to exhibit a wide range of neurological features and complications, posing the question of whether this virus is neurotropic [1]. Ranging from mild symptoms of dizziness, headache, dysgeusia, and anosmia, the neurological involvement can extend to severe central nervous system (CNS) dysfunction, including encephalopathy, cerebrovascular accidents [2], Guillain-Barré syndrome [3], meningoencephalitis, cerebral venous thrombosis, acute hemorrhagic necrotizing encephalopathy, and acute disseminated encephalomyelitis (ADEM) [4,5]. ADEM is a monophasic immune-mediated demyelinating CNS disorder that has been described as a post-infectious autoimmune disease primarily affecting children and, less commonly, adults, following a viral illness or, rarely, a vaccination [6]. Measles, rubella, varicella-zoster virus, influenza, Epstein-Barr virus, and herpes simplex virus are the usual viral infections preceding ADEM, as well as many other pathogens [7]. In 2004, Yeh et al. reported the first association between a human coronavirus (HCoV) and ADEM in a pediatric patient, whose cerebrospinal fluid (CSF) and nasopharyngeal swab were positive for coronavirus OC43 by polymerase chain reaction (PCR) [8]. In 2020, following the emergence of SARS-CoV-2, a growing body of evidence has demonstrated the correlation between these two entities. Numerous cases of ADEM, including the severe form of acute hemorrhagic leukoencephalitis (AHLE), following infection with this virus, have been reported [9]. Furthermore, cases of ADEM have also been described following the coronavirus disease 2019 (COVID-19) vaccination [10]. Contrasting the higher incidence of classical ADEM in children versus in adults, multiple reviews have reported a much higher incidence of this syndrome in adult age groups compared to the pediatric age group, following COVID-19 infection [9,11,12]. In the context of the still ongoing COVID-19 pandemic now concluding its third year, this case report describes one of the rare neurological complications of an infection with this virus, ADEM, and its successful medical management.

## Case presentation

This report describes the case of a male patient in his early 40s, unvaccinated for COVID-19, who presented to the emergency room (ER) with a 10-day history of dry cough, progressive dyspnea at rest, and intermittent fever. He had no medical or neurological history. Upon presentation, the patient was afebrile (36.8°C), hypoxic (89% by pulse oximeter on room air), and tachycardic (115 beats per minute). The Glasgow Coma Scale (GCS) score at admission was 15/15. He had no neurological deficits. Bilateral coarse crackles were heard mostly in the lower third of the chest upon auscultation of his lung fields. A non-enhanced CT scan of the chest, performed as an outpatient in another institution prior to presentation to our ER, revealed bilateral ground-glass opacities involving 60% of the lungs. The exact date of the CT scan was unobtainable. A nasopharyngeal PCR swab was positive for SARS-CoV-2. The patient was placed on supplemental oxygen of 10 L/minute by a face mask. Initial labs did not show a severe inflammatory response. Table 1 presents a summary of laboratory results and oxygen therapy parameters on select dates during the in-hospital stay.

**Table 1:** Laboratory variables and oxygen therapy on selected dates. WBC = white blood cells; Neut = neutrophils; Lymph = lymphocytes; Hb = hemoglobin; Plt = platelets; BUN = blood urea nitrogen; Cr = creatinine; Na = sodium; K = potassium; Cl = chloride; CO₂ = carbon dioxide; CRP = C-reactive protein; LDH = lactate dehydrogenase; PCT = procalcitonin; IL-6 = interleukin-6

Laboratory	Parameter	Admission	Day 5	Day 7	Day 14	Day 25	Reference ranges (SI)
Complete blood count	WBC	9,160/µL	10,950/µL	10,550/µL	22,730/µL	34,260/µL	4,000–11,000/µL
	Neut	84.9%	89.5%	92.1%	91.3%	90.6%	37–75%
	Lymph	10.7%	6.2%	5.2%	5.3%	5.1%	10–50%
	Hb	13.2 g/dL	13.4 g/dL	13.6 g/dL	11.7 g/dL	13.2 g/dL	13.5–18 g/dL
	Plt	184 000 /µl	336 000/µl	387 000/µl	246 000/µl	450 000/µl	140 000- 440 000
Chemistry and serum electrolytes	BUN	5.712 mmol/L	9.996 mmol/L	7.14 mmol/L	4.641 mmol/L	9.639 mmol/L	3.6–7.1 mmol/L
	Cr	97.26 µmol/L	70.74 µmol/L	70.74 µmol/L	61.89 µmol/L	44.21 µmol/L	53–106 µmol/L
	Na	134 mmol/L	139 mmol/L	136 mmol/L	140 mmol/L	141 mmol/L	136–145 mmol/L
	K	3.6 mmol/L	4.9 mmol/L	4.2 mmol/L	4.9 mmol/L	5 mmol/L	3.5–5 mmol/L
	Cl	101 mmol/L	100 mmol/L	100 mmol/L	106 mmol/L	103 mmol/L	98–107 mmol/L
	CO₂	23 mmol/L	23 mmol/L	24 mmol/L	25 mmol/L	30 mmol/L	20–28 mmol/L
Inflammatory markers	CRP	89 mg/L	78 mg/L	44 mg/L	206 mg/L	156 mg/L	Normal <10 mg/L
	Ferritin	806.8 ng/mL	1,368.9 ng/mL	780 ng/mL	1,713.97 ng/mL	1,447.1 ng/mL	30–220 ng/mL
	LDH	304 U/L	555 U/L	261 U/L	507 U/L	349 U/L	20–247 U/L
	D-dimer	234 ng/mL	509 ng/mL	571 ng/mL	10,505 ng/mL	-	0–500 ng/mL
	PCT	0.06 ng/mL	-	0.04 ng/mL	0.23 ng/mL	-	<0.1 ng/mL
	IL-6	21.29 pg/mL	-	-	-	-	0–7 pg/mL
Oxygen therapy		Face mask 10 L/minute	High-flow nasal cannula	High-flow nasal cannula	Invasive mechanical ventilation		

His medications at the time of admission included intravenous (IV) methylprednisolone 40 mg once daily, remdesivir 200 mg IV once started on the first day of admission, followed by 100 mg IV once daily for a total of five days, and prophylactic subcutaneous (SC) anticoagulation with enoxaparin 40 mg once daily. The patient’s oxygen demands increased on a daily basis, requiring the use of a high-flow nasal cannula (HFNC) at maximum settings of 100% oxygen and 60 L/minute flow, paralleling an increase in his inflammatory markers (Table 1). On the fifth day of admission, the patient received the first dose of tocilizumab (400 mg IV). He showed a moderate improvement demonstrated by a decrease in the oxygen flow of the HFNC to 15 L/minute, as well as a decrease in his markers (Table 1). On the ninth day of admission, his respiratory status started declining again (oxygen flow was increased to 60 L/minute). A second dose of tocilizumab (400 mg IV) was given, and he was started on piperacillin/tazobactam empirically at a dose of 4.5 g IV every six hours. No improvement was noted. He became febrile (39.9°C) and continued to gradually worsen.

On the 14th day of admission, the patient developed acute respiratory distress syndrome (ARDS) and type II acute respiratory failure. He was intubated and placed on invasive mechanical ventilation. His peri-intubation labs were as follows: white blood cell (WBC) count 22,730/µL, C-reactive protein (CRP) 206 mg/L, procalcitonin (PCT) 0.23 ng/mL, ferritin 1,713.97 ng/mL, D-dimer 10,505 ng/mL, and lactate dehydrogenase (LDH) 507 U/L (Table 1). Furthermore, his viral pneumonia was complicated by multidrug-resistant (MDR) *Acinetobacter boumanii* and methicillin-resistant *Staphylococcus aureus *(MRSA) nosocomial pneumonia at the time of respiratory failure. Targeting these two bacteria, piperacillin/tazobactam was stopped after five days, and the patient was started on colistimethate sodium, at a dose of 4.5 million units IV every 12 hours, combined with linezolid, at a dose of 600 mg IV every 12 hours, both continued for 14 days. His fever resolved 48 hours following this new regimen.

**Figure 1:**
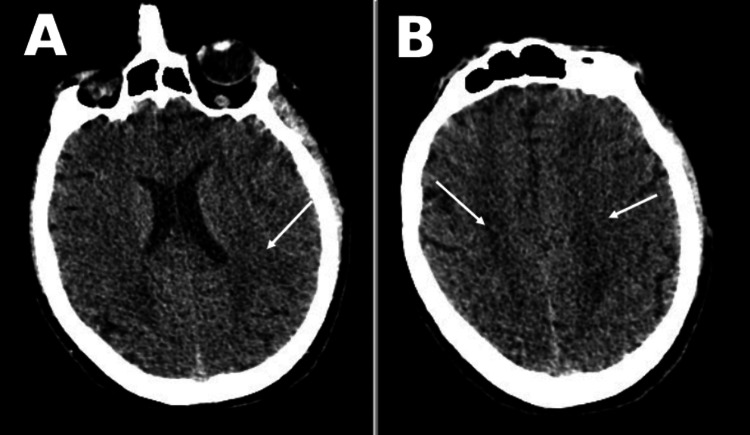
(A, B) Non-enhanced CT scan of the brain showing moderately decreased attenuation of the periventricular and the semiovale white matter (arrows).

**Figure 2:**
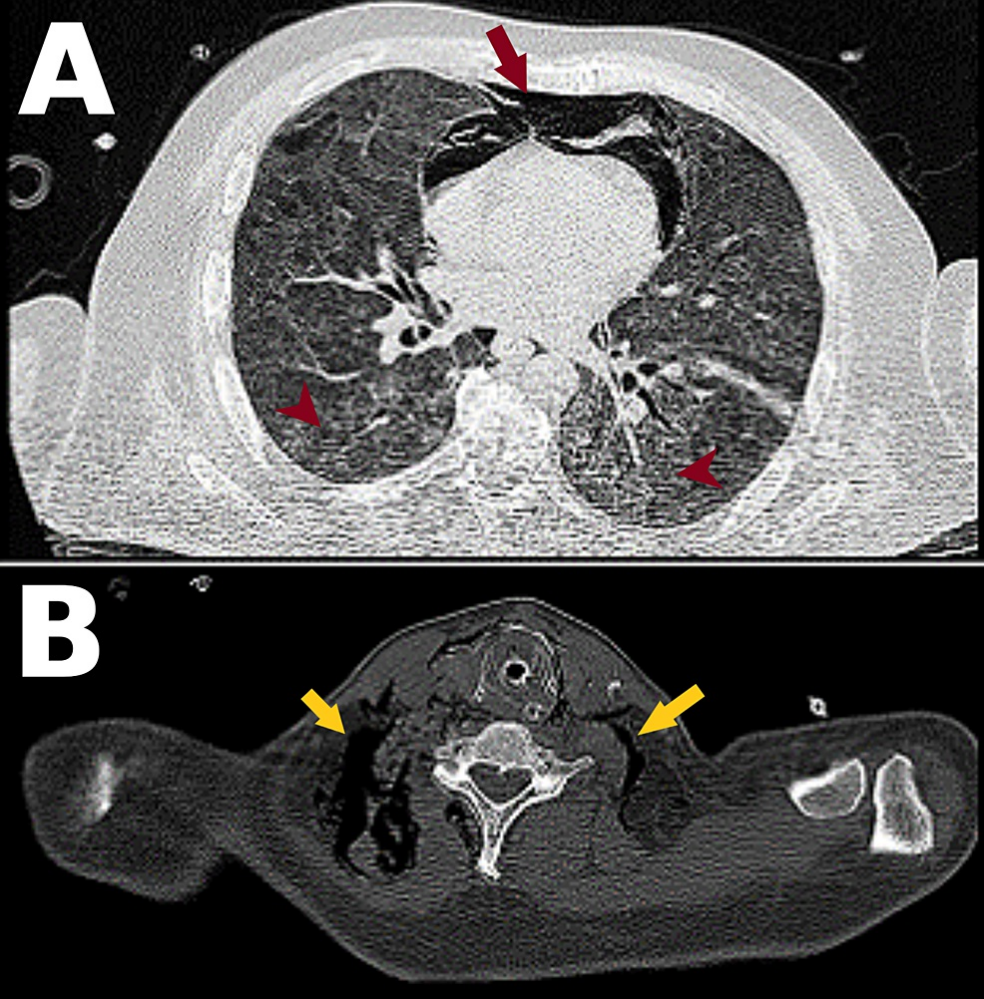
Non-enhanced CT scan of the chest. A: Lung view showing diffuse bilateral ground-glass opacities (red arrowheads) and pneumomediastinum (red arrows). B: Mediastinal view showing subcutaneous emphysema (yellow arrows).

Prior to intubation, the patient had no focal neurological deficits or signs of encephalopathy. Following intubation, the patient was kept sedated using midazolam by continuous infusion and cisatracurium for eight days. As his clinical condition and biochemical panel started improving on mechanical ventilation and antibiotics, sedation was stopped in an attempt to wean; however, 72 hours after stopping sedation, the patient remained non-responsive. His GCS score was 5T (eye-opening: 4, motor response: 1). He was not alert, both pupils were equal, medium-sized and located, and reactive to light. All brainstem reflexes were preserved. The motor power was 0/5 in all extremities, muscle tone was flaccid, Babinski and deep tendon reflexes were absent, and there was no response to painful external stimuli in any extremity. An urgent non-enhanced CT scan of the brain and chest, performed on the 24th day of admission, revealed moderately decreased attenuation of the periventricular and semiovale white matter (Figure 1) and severe pneumomediastinum and emphysema, as well as severe diffuse bilateral ground-glass opacities (Figure 2).

At this point, the differential diagnosis included encephalitis, metabolic/septic encephalopathy, or non-convulsive status epilepticus. During this period, the patient became febrile, with an elevated WBC count and CRP level (Table 1, day 25). Thus, a septic encephalopathy was still suggested, and the patient was continuing the course of antibiotics. A lumbar puncture (LP) showed clear and colorless CSF with no leukocytes, 400 red blood cells, 23 mg/dL protein (reference range = 12-60 mg/dL), and 3.9 mmol/L glucose (reference range = 2.2-3.9 mmol/L). Gram stain, bacterial, and fungal cultures were all negative, thus ruling out meningoencephalitis. On grounds of the clear CSF, a PCR viral panel was not performed. Serum myelin oligodendrocyte glycoprotein antibody (anti-MOG) was negative. His serum sodium remained within normal limits throughout the admission. Serum ammonia level was elevated (157.9 µmol/L, reference range = 0-35.2 µmol/L), without any alteration in liver function tests. Lactulose and rifaximin were initiated; however, the treatment for hyperammonemia metabolic encephalopathy did not lead to any amelioration of the patient’s level of consciousness and was eventually ruled out as the primary cause.

**Figure 3:**
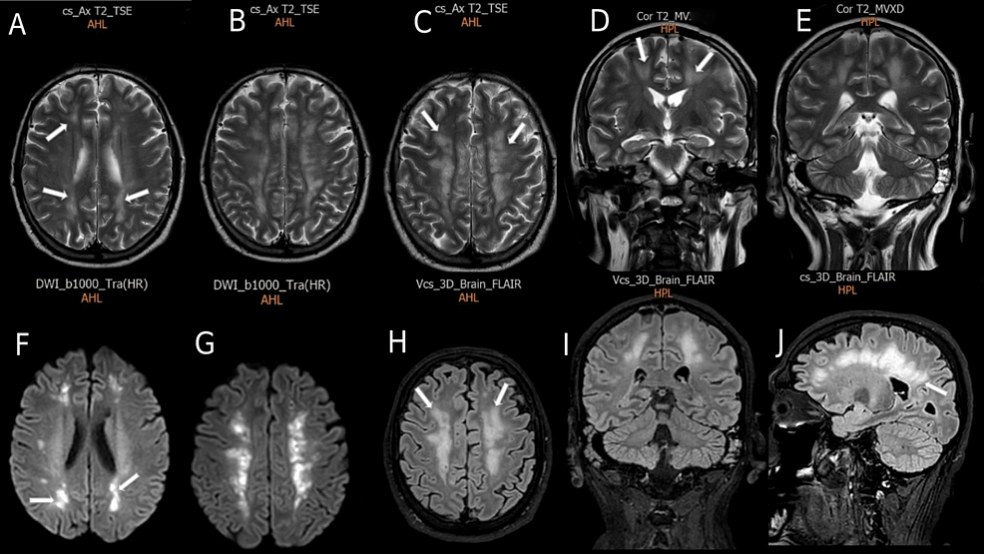
First MRI of the brain showing multiple bilateral periventricular and semiovale punctate and oval lesions (white arrows) with surrounding edema that show hyperintensity on T2W images (A, B, C, D, E) and FLAIR images (H, I, J) with diffusion restriction (F, G). T2W = T2-weighted; FLAIR = fluid-attenuated inversion recovery

MRI of the brain with angiography/venography, without including the spinal cord, was done on the 28th day of admission (Figure 3). It demonstrated multiple periventricular white matter punctuate and oval lesions with surrounding edema, as well as in the bilateral centrum semiovales, showing signal hypointensity on T1-weighted (T1W) images, hyperintensity on T2-weighted (T2W) images, and fluid-attenuated inversion recovery (FLAIR) images, with diffusion restriction. These MRI findings were consistent with a new demyelinating disease, and ADEM was highly suspected.

An electroencephalogram (EEG) was not done due to technical issues; hence, as a precautionary measure, it was decided to place the patient on sodium valproate at a dose of 400 mg IV twice daily. No change in his mental status was observed. At this time, the patient had been off antibiotics for the previous three days, after the course was completed. However, his leucocyte count, CRP, and PCT level started to increase (WBC 11,030/µL, CRP 178 mg/L, PCT 1.09 ng/mL). Prior to any decision on starting high-dose steroids, antibiotics were initiated empirically to stunt the infectious process, which did not resolve nor improve the patient’s encephalopathy. Hence, ADEM treatment was deferred for seven days until the infectious profile was contained. Then the patient was started on 1 g IV methylprednisolone once daily for five days, directly followed by intravenous immunoglobulin (IVIG) therapy (40 g) for three days.

At this time, a tracheostomy procedure was performed, and the patient was breathing room air. Near the end of the IVIG course, the patient was already showing a grimace at painful stimulation. Gradually, he started following movements with his eyes, became clinically aware of his surroundings, and his speech abilities were being restored. He recognized family members, and bodily motor and sensory powers returned, albeit very slowly, starting with faint movements of his fingers and toes. Physiotherapy was initiated as soon as clinically possible. MRI of the brain and spinal cord was repeated 18 days following the first MRI. It demonstrated the same lesions with the same characteristics, unchanged from the first MRI. No lesions were detected in the spinal cord.

**Figure 4:**
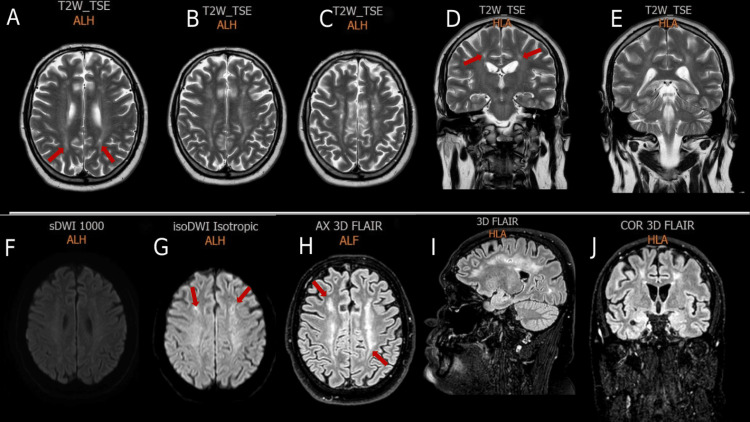
Third MRI of the brain showing a decrease in the size of the previous hyperintense lesions (red arrows) on T2W sequences (A, B, C, D, E), diffusion-weighted sequences (F, G), and FLAIR sequences (H, I, J). T2W = T2-weighted; FLAIR = fluid-attenuated inversion recovery

Overall, the patient’s course was complicated by severe sepsis, ARDS requiring high positive end-expiratory pressure ventilation (PEEP), pneumomediastinum and subcutaneous emphysema, atrial fibrillation, severe erosive gastritis, and anemia requiring blood transfusions. He underwent tracheostomy and gastrostomy procedures and was discharged home after a total of 50 in-hospital days. One month after discharge, the patient was using a wheelchair. Two months after discharge, the tracheostomy and gastrostomy tubes were removed. He transitioned from using a walker about four months following discharge, to using a cane one month after that, and to freely self-ambulating one month after. His neurological examination demonstrated residual right-sided foot drop. The third MRI was done 109 days after the second MRI, which showed a decrease in the size of the previously mentioned lesions, without the development of new lesions (Figure 4).

## Discussion

There are two possible pathways that have been proposed for the entry of SARS-CoV-2 into the central nervous system: the hematogenous route, where the virus invades the CNS by infecting cells of the blood-brain barrier, and the neuronal pathway, where the virus propagates to the CNS by axonal transport after infecting peripheral nerves [13]. The characteristic pathological feature of ADEM consists of perivenous areas of demyelination accompanied by the infiltration of various types of inflammatory cells [14]. The pathogenic mechanism for the development of ADEM following viral illness is still unclear; however, molecular mimicry has been suggested as a likely process [15,16].

ADEM typically presents with multifocal neurological symptoms and encephalopathy accompanied by the appearance of multifocal demyelination on neuroimaging [7]. A systematic review of COVID-19-related ADEM conducted by Wang et al. reported variable symptoms ranging from encephalopathy, headache, fever, and seizures to neurological deficits, including gait ataxia, hemi and tetraplegia, focal sensory and motor deficits, and hyporeflexia or areflexia, with a mean duration of 24.7 days from symptomatic COVID-19 till the diagnosis of ADEM [11]. The occurrence of COVID-19-related ADEM does not always follow a severe COVID-19 infection, as shown in the review by Zelada-Ríos et al., where five cases reported symptoms, all of which were mild or asymptomatic [12]. Another review article by Manzano et al. found that 67% of the reviewed cases had severe COVID-19 disease and required intensive care admission [9].

Findings on MRI typically illustrate white matter multiple, bilateral, asymmetric, poorly defined lesions of variable sizes that best appear hyperintense on T2W and FLAIR images [17,18]. Lesions often involve the periventricular and the subcortical white matter, but may also involve the gray matter, such as the cortex, basal ganglia, and thalamus [16]. Involvement of the spinal cord, which could include hyperintense enhancing lesions that may be accompanied by edema, has been reported in 11-28% of children [18]. The diagnosis of ADEM necessitates the precise exclusion of other demyelinating CNS diseases, the entity being relatively rare. Hence, a diagnosis of ADEM requires the fulfillment of the following criteria: a first polyfocal CNS event with a presumed demyelinating cause, encephalopathy, unexplained by other causes, characteristic brain MRI abnormalities during the acute three-month phase, and the absence of new clinical or MRI findings three months or more following the initial event [17,19].

Our patient had critical COVID-19 with ARDS requiring mechanical ventilation, high PEEP, and sedation. The first symptom pointing to a complicating neurological disease was the failure to awaken from sedation. The duration from contracting COVID-19 to the diagnosis of ADEM was 38 days. The patient demonstrated encephalopathy, portrayed by a decrease of GCS, with an extensive motor and sensory disease, and was quadriplegic. Seizures could not be ruled out as a symptom due to the absence of an EEG at the time. His LP did not display any abnormality. Serum anti-MOG antibody was negative, thus excluding myelin oligodendrocyte glycoprotein antibody-associated disease. His MRI showed characteristic demyelinating white matter lesions pointing to ADEM, without any spinal cord involvement. After hyperammoniac metabolic encephalopathy and meningoencephalitis were ruled out, the treatment of his sepsis did not ameliorate the encephalopathy, and, eventually, the latter was considered to be a criterion for ADEM. The extent of the neurological involvement raised the question of possibly accompanying other neurological entities along with ADEM. Guillain-Barré syndrome was discussed as a potential compounding event, as well as the possible neurotoxic effect of colistimethate sodium, which was used to treat *A. boumanii* pneumonia. The complete workup was, however, not performed. The patient was treated with both pulse steroids and IVIGs with a good response that was noted toward the end of the IVIG treatment. A follow-up MRI more than three months after the event did not demonstrate any new lesion, and, therefore, our case has met the criteria for ADEM. Coupled with extensive prolonged physiotherapy, the patient had a brief morbidity period of six months in total and an overall good prognosis.

## Conclusions

We report a case of critical COVID-19 complicated by severe neurological dysfunction, and MRI findings suggestive of ADEM. After excluding other entities, our patient was treated for ADEM and showed a positive response and almost complete resolution of neurological deficits. Our case serves to add to the bulk of data relating neurological involvement to SARS-CoV-2 infection.
